# Design Methodology of an Equalizer for Unipolar Non Return to Zero Binary Signals in the Presence of Additive White Gaussian Noise Using a Time Delay Neural Network on a Field Programmable Gate Array

**DOI:** 10.3390/s131216829

**Published:** 2013-12-06

**Authors:** Santiago T. Pérez Suárez, Carlos M. Travieso González, Jesús B. Alonso Hernández

**Affiliations:** Institute for Technological Development and Innovation in Communications, University of Las Palmas de Gran Canaria, Las Palmas de Gran Canaria 35017, Spain; E-Mails: ctravieso@dsc.ulpgc.es (C.M.T.G.); jalonso@dsc.ulpgc.es (J.B.A.H.)

**Keywords:** equalizer, AWGN, neural network, FPGA, floating point, fixed point, Matlab, Simulink, System Generator, ISE, 84.40.Ua, 89.70.-a, 89.70.Hj, 89.20.Ff, 07.50.Qx, 84.30.Sk, 07.50.Hp, 07.05.Mh

## Abstract

This article presents a design methodology for designing an artificial neural network as an equalizer for a binary signal. Firstly, the system is modelled in floating point format using Matlab. Afterward, the design is described for a Field Programmable Gate Array (FPGA) using fixed point format. The FPGA design is based on the System Generator from Xilinx, which is a design tool over Simulink of Matlab. System Generator allows one to design in a fast and flexible way. It uses low level details of the circuits and the functionality of the system can be fully tested. System Generator can be used to check the architecture and to analyse the effect of the number of bits on the system performance. Finally the System Generator design is compiled for the Xilinx Integrated System Environment (ISE) and the system is described using a hardware description language. In ISE the circuits are managed with high level details and physical performances are obtained. In the Conclusions section, some modifications are proposed to improve the methodology and to ensure portability across FPGA manufacturers.

## 1. Introduction

Artificial Neural Networks (ANNs) have been widely used as identifiers of patterns [1], and their advantages and benefits are well known. This paper exposes a design methodology for designing an ANN as an equalizer for a binary signal. The signal is a unipolar Non Return to Zero (NRZ) with Additive White Gaussian Noise (AWGN) [2]. Firstly, the system is modelled in floating point format using Matlab [3]. Afterward, a design methodology is described for a Field Programmable Gate Array (FPGA) using fixed point format [4].

The FPGA design is based on the System Generator from Xilinx [5]. Xilinx is one of the most important FPGA manufacturers, and System Generator is a design tool over Simulink of Matlab [6]. Simulink is a graphical environment Matlab design tool. In Simulink designs are described in the form of block diagrams and it has utilities for displaying and analysing the simulations. System Generator allows one to design in a fast and flexible way. It uses a low level of circuit details, so the simulations are fast, and the functionality of the system can be fully tested. System Generator can be used to check the architecture and to analyse the effect of the number of bits on the system performance. Furthermore, it gives an approximate estimation of required hardware resources. Finally, physical performances are extracted with the Xilinx Integrated System Environment (ISE) [7].

In this regard there have been several studies on ANN over FPGA for real time processing. Some of them focused on baseband signals, and are used as receptors [8] or as equalizers [9,10]. In the same way, others studies are for band pass signals [11]. The equalization can be performed to minimize distortion and noise introduced in the channel. With modulated signals such systems can also be used for identifying the modulation type [12]; obviously, these studies are restricted to certain values of carrier frequencies and certain types of modulation. In multiuser communication ANNs are used to identify and synchronize the channel or to make demodulation [13–15]. Other studies have been developed on a very specific scenario [16]. In any case, few have been developed on FPGA [17], generally these studies use numerical floating point format on a personal computer. Many of these studies focus on the development of new architectures of ANNs [18] or new training methods [19].

When the rate of the input signal increases the ANN implemented in a computer in floating point format cannot operate in real time. For decreasing the response time the ANN should be passed to a digital circuit, normally in fixed point format. The reason is that floating point arithmetic in a digital device needs a lot of hardware resources and power, without substantial improvement in speed. Besides, with the digital device the volume and the power consumption will decrease.

One alternative is to use an Application Specific Integrated Circuit (ASIC). The ASIC has low area occupation, low power consumption and high speed, but its disadvantages are: high price, difficult debugging and verification, long time to market, the fact that it does not allow reprogramming and has high non-recurring engineering costs. For these reason, ASIC is undesirable to develop prototypes where the number of units to be produced is small.

On the other hand, Digital Signal Processors (DSPs) can be used, which are cheaper than ASICs. DSPs reach higher clock frequencies, but the data rate that can be processed is limited because of the parallelism of the data, the size and format of the data, and the pipelined are fixed. All this is imposed by its predetermined architecture.

Finally, the use of Field Programmable Gate Arrays (FPGA) has several advantages: low price, no non-recurring engineering costs, minimum development time, ease of debugging and verification, short time to market, high data parallelism, flexible data format and flexible pipelined structure. Although the clock frequency is not as high as in DSPs, with the above characteristics an increase in the data rate can be achieved. Moreover FPGAs have higher power consumption, but they are appropriate for individual prototypes because FPGAs can be reprogrammed by the designer.

## 2. The Equalizer System

**Figure 1.**
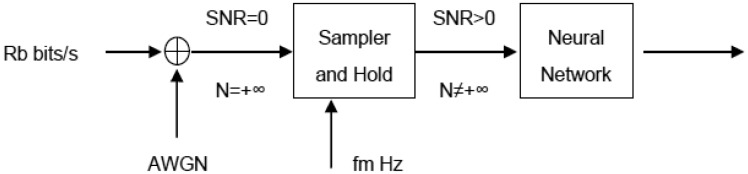
Proposed model.

This study focuses on a binary unipolar NRZ signal, and the digital cero (“0”) and digital one (“1”) have the same probability (Figure 1). That is, the “1” and “0” are respectively represented by +*A* and 0 volts (or amps) during a bit time (*Tb* seconds). The bit rate value is *Rb* = *1*/*Tb* bits per second. For simplicity and without loss of generality it may be assumed that +*A* is equal to *1*. It is assumed that the signal is affected by additive AWGN. The received NRZ signal has infinite bandwidth and does not suffer distortion, although its higher spectral components are near the zero frequency. The AWGN also has infinite bandwidth and its power spectral density is uniform, its power is infinite, and therefore the Signal Noise Relation (SNR) is zero. The AWGN is not bounded in amplitude, although very large values are unlikely as indicated its Gaussian probability density function. The input signal of the Sampler and Hold block is analogue and continuous.

In summary, it is assumed that the signal has been transmitted over a channel with infinite bandwidth which adds AWGN. The received signal is sampled each *Tm* seconds; the sample frequency is *fm* = *1*/*Tm* Hz. An integer number of samples will be taken in each bit interval. Each sample of the sampled signal is composed of the data signal component plus the noise component. The component of the sampled noise is additive Gaussian with zero mean value. The noise power at the output of the sampler is finite and is given by the variance, which is the same as the square of the typical deviation. The SNR in the sampler output is a nonzero finite value. The sampler output is a discrete time signal, and it can be introduced into a digital system through a convenient quantification. The output of the sampler can be introduced into a discrete time digital system.

One objective of this study is to check if a Time Delay Neural Network (TDNN) can be used as a preamplifier or equalizer; increasing the output Signal to Noise Relation. Furthermore, it is proposed a design methodology over a FGPA for the TDNN. The tool used to simulate the system in floating point format was Matlab and Simulink, and especially the Neural Network Toolbox was used [20].

**Figure 2.**
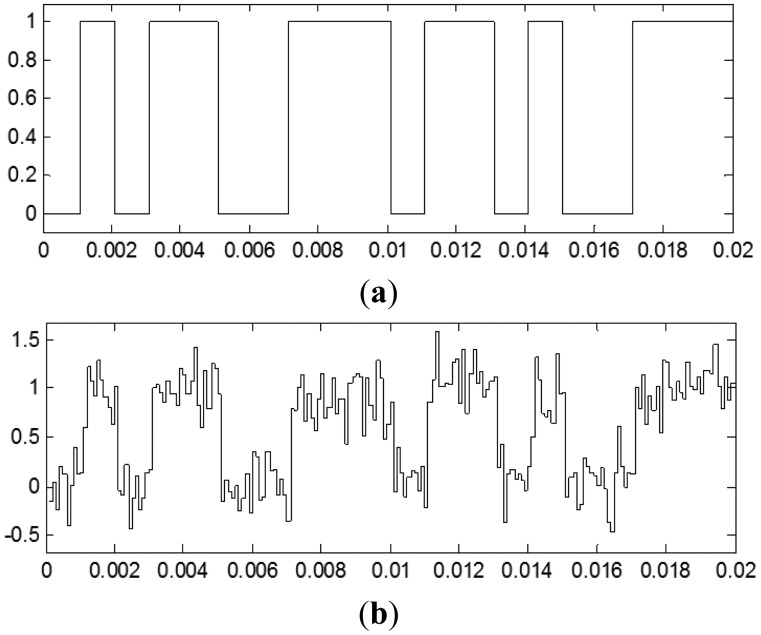
(**a**) Original data signal and (**b**) sampled received signal.

Initially the bit rate (*Rb*) was set to one kilobit per second and ten samples were taken per bit (*n* = 10), therefore the sampling frequency (*fm*) was set to 10 kHz. The value of the bit rate has not transcendence, the important parameter is the number of samples per bit. In the final system the bit rate can increase as much as the technology permits, according to the maximum clock frequency. Figure 2 shows the original data signal and the sampled received signal with +10 dB of SNR.

## 3. The Floating Point Modelling

**Figure 3.**
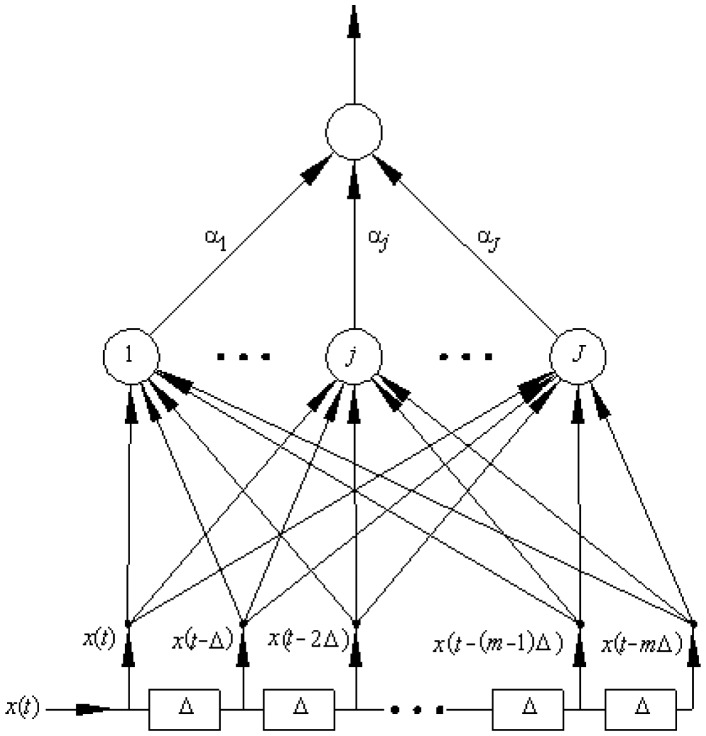
Time delay neural network.

Figure 3 shows a Time Delay Neural Network (TDNN), it is a neural network which includes input cascaded delay elements. In this case, each element delays the signal a sampling interval (*Tm* seconds). For processing *n* samples, (*n* – 1) delay cells are necessary. This architecture has a transitory period for the first input symbol until the first *n* samples arrive. Without the delay cells the system is a Multilayer Perceptron Neural Network type.

The question is whether this TDNN will improve the SNR of the sampled signal. This TDNN is trained with its input noisy sampled signal and the target is the original data signal. The signal received at the input of the sampler is called *r*(*t*), which is equal to the data signal *d(t)* plus noise signal *n(t)*; that is, *r*(*t*) = *d*(*t*) + *n*(*t*). At a given time, called *t_0_*, the delay elements of the neural network store the samples *r*(*t_o_* – *kTm*), where *k* is equal to *0, 1, 2*, …, *9*. For these values the target for the neural network training is *d(t_o_)*, the original data value in *t_0_*. The observation interval is *Tb* seconds.

Initially to train, validate and test the neural network a sequence of 1,000 random bits with +10 dB of SNR was used. Only one hidden layer with five neurons was used, so the neural network size can be denoted as 10/5/1.

**Figure 4.**
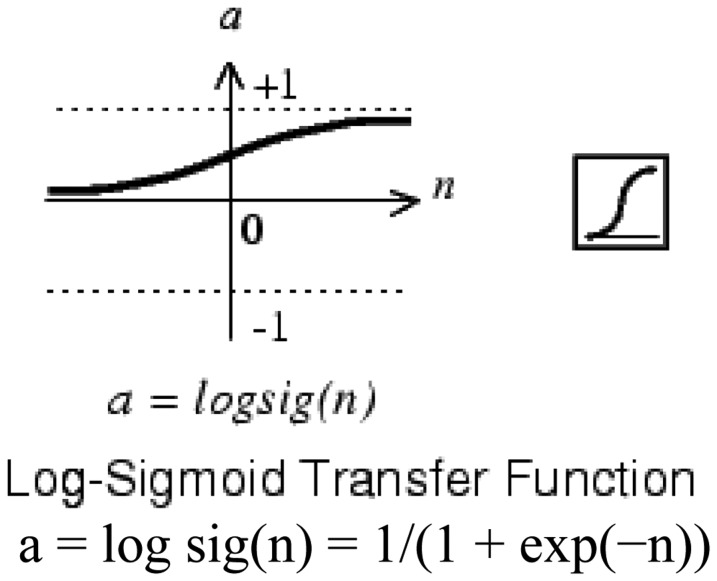
Log-sigmoid transfer function.

Typically the number of neurons in the intermediate layer is initially the geometric or arithmetic average between the inputs and outputs. As transfer function the “logsig” type was used in all neurons (Figure 4). The Levenberg-Marquardt algorithm was used for training with the Mean Squared Error (MSE) function. In this process, the Neural Network Toolbox of Matlab was used, and 80 percent of the samples were taken for training, 10 percent for validation and 10 percent for testing.

**Figure 5.**
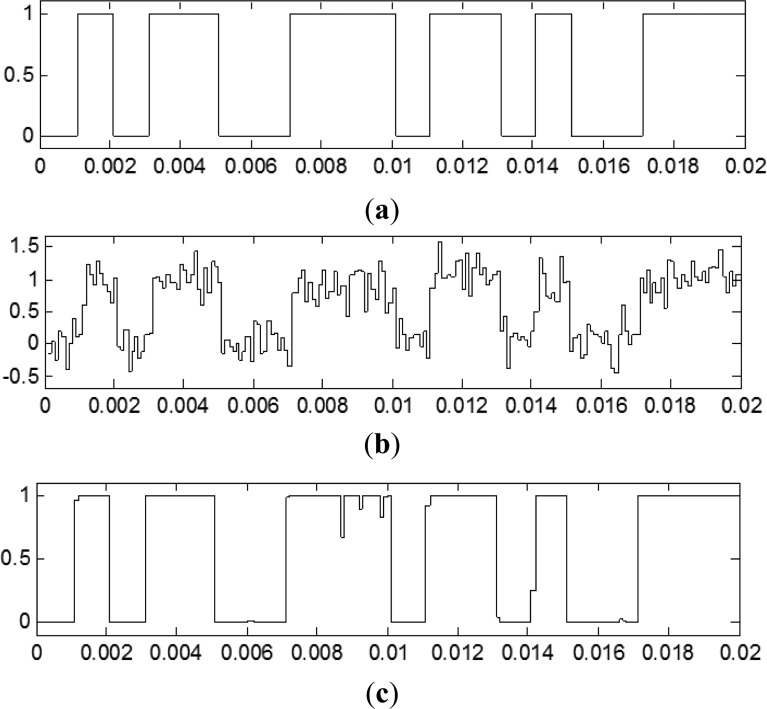
(**a**) Original data signal, (**b**) noisy sampled signal with +10 dB of SNR and (**c**) TDNN output.

Secondly, the neural network was tested with another 1,000 random bits for the same SNR (+10 dB). It can be said that the testing SNR was +10 dB. Original data, sampler output and TDNN output are shown in Figure 5. It can be observed that the SNR has improved, and it will be measured below.

The TDNN restricts the output signal to (0,1) interval because the output neuron has as transfer function a “logsig” type. The error in the output is bounded to 1. It should be noted that the signal power in the input and output have the same value. This is due to the waveform obtained in the output, because of the target values specified on training.

**Figure 6.**
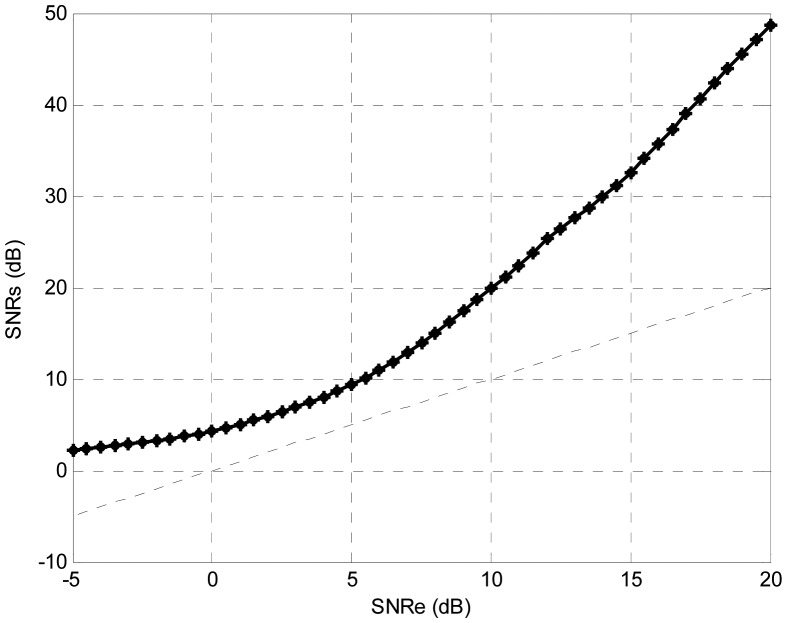
Output SNR *versus* input SNR in TDNN.

Finally, the TDNN trained with +10 dB of input SNR, was tested with different values of the input SNR. The testing SNR was varied from −5 dB to +25 dB in 0.5 dB increments. For each testing SNR 1,000 random bits were simulated. Figure 6 shows the output SNR *versus* input SNR, where the straight line where the output would be equal to the input is marked. The output SNR is always greater than the input SNR. It should be noted that the curve depends on the training, but this is the typical shape obtained.

The high number of parameters involved in the TDNN design should be emphasized. First of all, other neural network architectures are possible, some of them have signal feedbacks from the output to the input and besides the number of feedback samples can be varied. This study is focused on a TDNN without feedback. Once the architecture of the neural network has been fixed the design parameters are the number of intermediate layers, the number of neurons in each layer, the transfer function used in neurons, the training algorithm, the error function used in training, the observation interval duration, the training SNR, the testing SNR, the duration of the signal used for training, validation and testing, and the method of splitting the sampled signal for training, validation and testing.

### 3.1. The Number of Layers and Neurons

**Figure 7.**
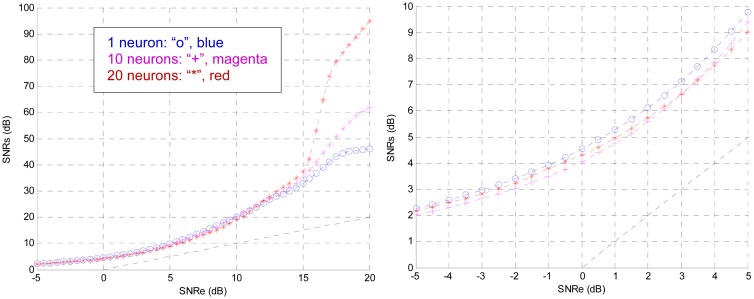
Effect of the number of neurons in the hidden layer on the output SNR.

Hereafter, the effects of these parameters are analysed. To observe the effect of the number of intermediate layers were tested configurations with two and three hidden layers, while the rest of the parameters remained as in the previous section. None of them trained successfully. Therefore, the architecture is fixed with a single intermediate layer. Then the effect of the number of neurons in this layer was analysed; for this purpose it was varied from 1 to 20. Figure 7 shows the curves for 1, 10 and 20 neurons, respectively

For high SNR in the input (>15 dB), one neuron in the middle layer gets high values of the output SNR (>30 dB). For low input SNR the curves converge. At this point the designer must establish a design criterion. It can be set to one neuron in the hidden layer if SNR values are good enough for the application; if not, it should be increased. For describing the method one neuron is fixed in the hidden layer. This is the smallest architecture, therefore it minimizes the area, the response time and power consumption. It must be emphasized that the results depend on the training, but these are the standard curves obtained. Henceforth, the system will have a single neuron in its intermediate layer.

### 3.2. Effect of the Transfer Functions

**Figure 8.**
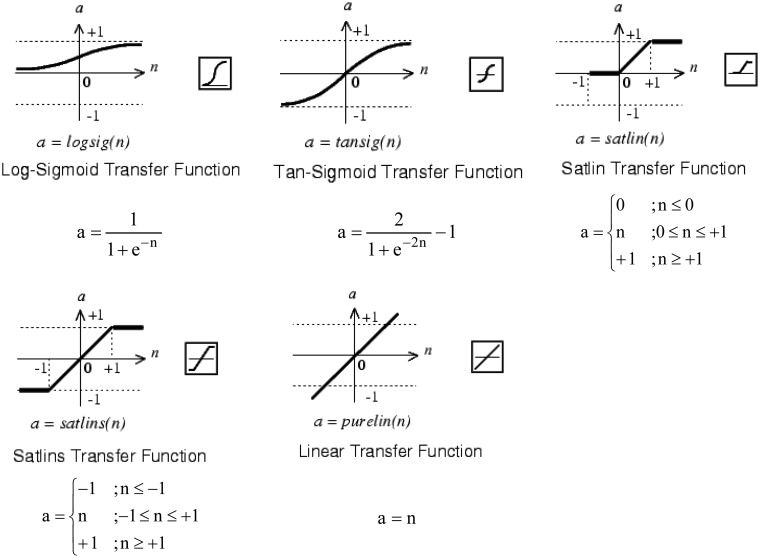
Transfer functions tested in the intermediate layer.

**Figure 9.**
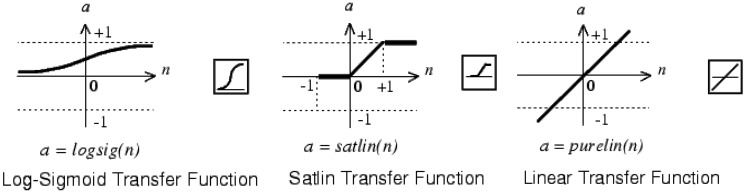
Transfer functions tested in output neuron.

At this point the effect of different transfer functions can be checked. The other parameters were held as in the previous configuration. The transfer functions must be increasing ones and differentiable except at specific points. The function chosen can have impact in the hardware implementation. In the intermediate layer the transfer functions shown in Figure 8 were checked. Since the unipolar output signal is between 0 and 1, three types of functions were tested in the output neuron (Figure 9).

**Figure 10.**
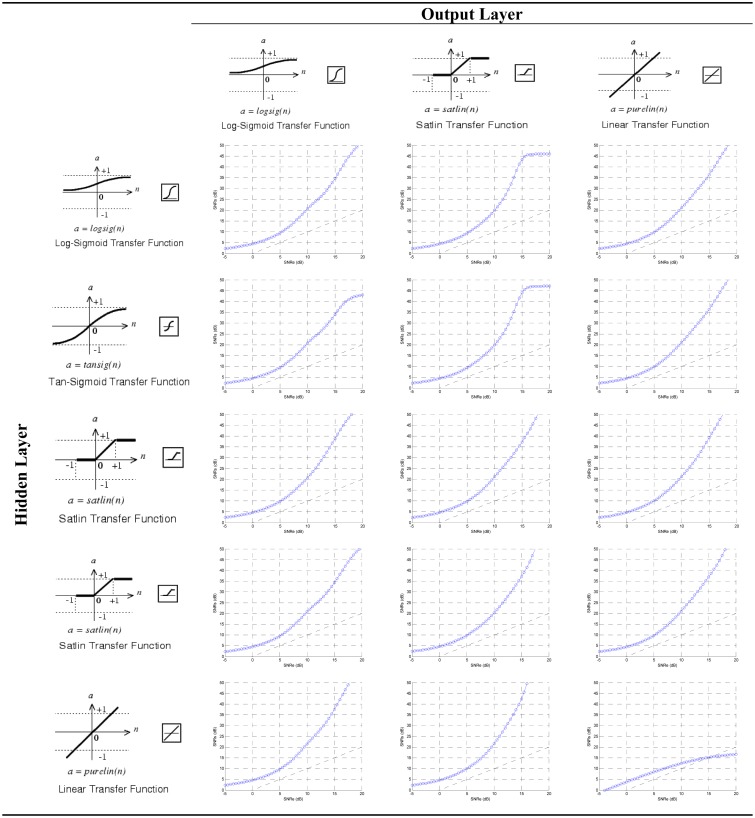
Transfer functions checked in the neural network.

The combinations shown in the Figure 10 were simulated; in fact, the Log-Sigmoid Transfer Function for all neurons already has been tested in the previous section. The curves obtained depend on the training, the figure shows typical shapes.

In order to facilitate the design of the transfer function on a FPGA the cases in which the intermediate layer has a Log-Sigmoid or Tan-Sigmoid Transfer Function were rejected (the first two rows of the Figure 10). Both cases have similar shape for the output SNR. Such types of functions are nonlinear and must be implemented using a method of approximation: memories, piecewise linear, polynomial, *etc*.

When the intermediate layer has a Satlin or Satlins Transfer Function (third and fourth row of the Figure 10) the output SNR is similar to previous cases. Since these functions are piecewise linear their hardware implementation is simpler than that of the nonlinear functions, they need less hardware resources, have less power consumption and less time delay. Basically they can be designed with comparators and multiplexers, or using adders and the absolute value operator. Moreover, these implementations will not introduce approximation errors.

In the last row of the Figure 10 the Linear Transfer Function is used for the hidden layer, which is the identity function. The design of this function is trivial, and the output is equal to the input. It can be implemented with a short circuit with no approximation error. Therefore, this implies the lowest hardware resources, lowest power consumption and non-significant time delays. In the output neuron Linear Transfer Function is discarded by low output SNR. Log-Sigmoid and Satlin Transfer Functions produce similar output SNR. For the above reasons the Satlin Transfer Function was chosen for the output neuron. Thereafter the TDNN will have nine delay cells for processing 10 samples of the received signal. The hidden layer has one neuron with the Linear Transfer Function and the output neuron has the Satlin Transfer Function. For this modelling the Matlab Neural Network Toolbox was used.

### 3.3. Algorithm and Error Function Used for Training

For the above conditions, twenty training algorithms were tested. The Conjugate gradient backpropagation with Polak-Ribiere updates training algorithm was set because of the output SNR obtained, the limited number of iterations and the speed of convergence.

**Figure 11.**
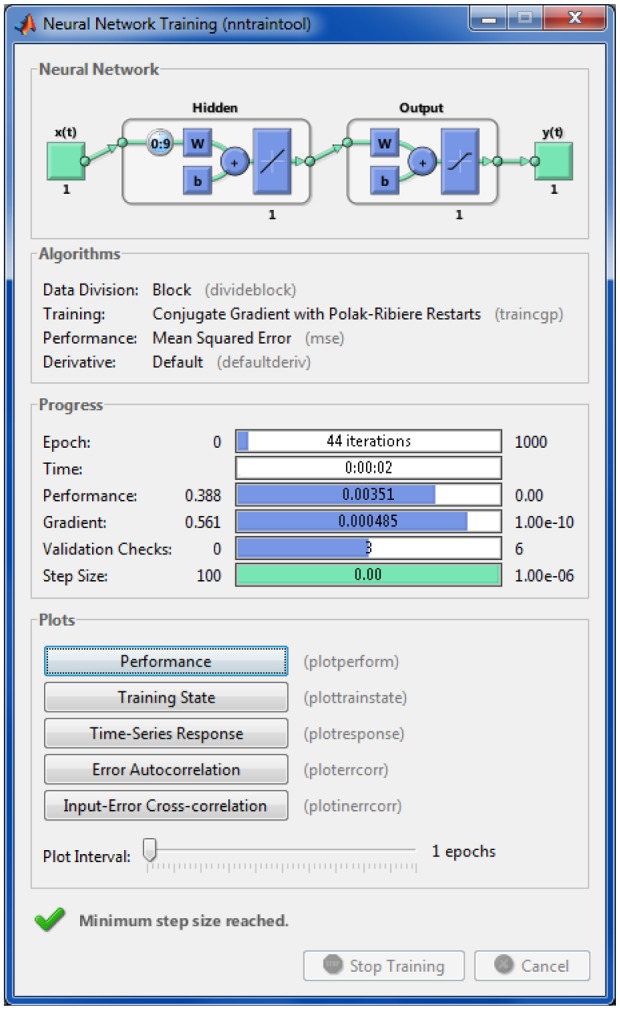
Matlab Neural Network Toolbox training window. Matlab has a bug in the output neuron drawing the unipolar Satlin Transfer Function as bipolar.

So far, the Mean Squared Error function has been used for the training algorithms. Under the conditions of the previous section, including the mentioned training algorithm, the effect of the error function was analysed. There are four functions available: mean squared error, mean absolute error, sum squared error and sum absolute error. The error function is not critical for the training; despite this, the Mean Squared Error function is used for the small improvement for low input SNR. Figure 11 shows the training window, which also details this architecture.

### 3.4. The Observation Interval Duration

**Figure 12.**
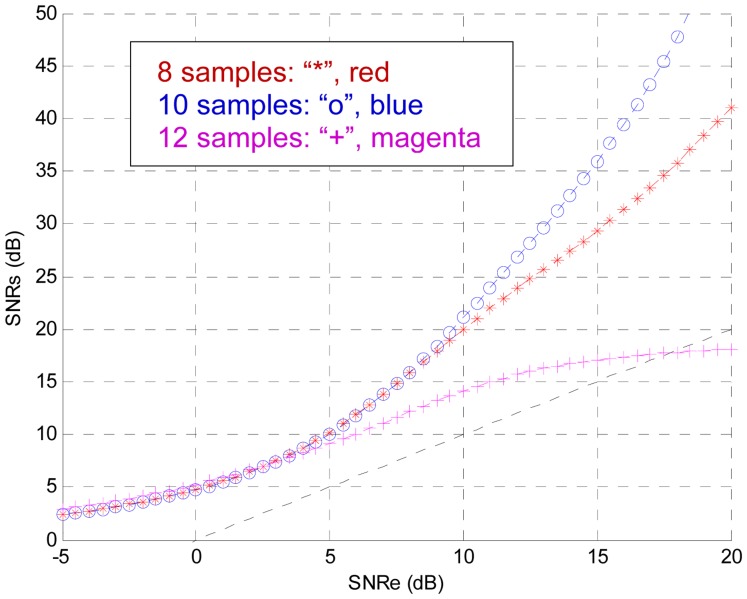
Effect of the observation interval.

In this section the effect of the size of the observation interval is analysed. The rest of the parameters have the same configuration of the previous section. For this purpose 10 samples per bit are taken, the sampling frequency is 10 kHz and the training SNR is +10 dB. The number of samples processed by the TDNN is varied between 1 and 20. The best result is for 10 processed samples. Figure 12 shows the testing SNR for 8, 10 and 12 processed samples, respectively.

These simulations justify the use of an observation interval of *Tb* seconds. Obviously, if less than 10 samples per bit are taken the system is deteriorated, the performance can be improved by increasing the sampling frequency, but this can be a design constraint.

If the observation interval is longer than *Tb* seconds, when the last arrived sample coincides with the beginning of a bit then the two previous bits are being processed. The last received sample should force the decision in the TDNN output. In this case, the older bit is an unnecessary input for the system. For a fixed number of samples processed, if the observation interval is smaller than a bit duration (*Tb* seconds) then the sampling frequency is increased, but this can be a design constraint.

### 3.5. Signal to Noise Relation for Training

**Figure 13.**
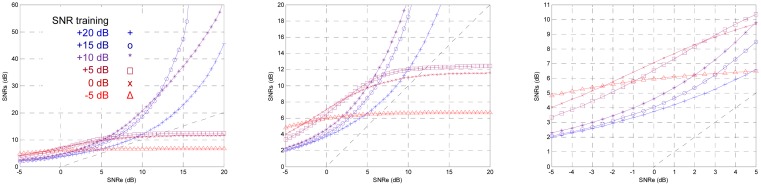
Effect of the training SNR from −5 dB to +20 dB.

The noise power is one of the most important parameters in the TDNN design. It was tested by sweeping the training SNR from −5 dB to +20 dB in 1 dB steps. Afterwards, the testing SNR was varied from −5 to +20 dB in 0.5 dB steps for each training SNR. In Figure 13 the curves for different training SNR are shown. It underscores the importance that:

- Training with −5 dB causes the best testing SNR for −5 dB,
- Training with 0 dB causes the best testing SNR for 0 dB,
- Training with +5 dB causes the best testing SNR for +5 dB,
- Training with +10 dB causes the best testing SNR for +10 dB,
- Training with +15 dB causes the best testing SNR for +15 dB,
- But training with +20 dB does not cause the best testing SNR for +20 dB; in fact, the best testing SNR for +20 dB is for +15 dB training SNR.

**Figure 14.**
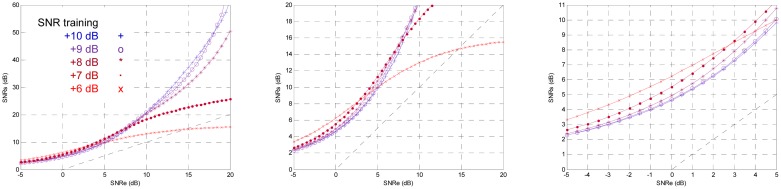
Effect of the training SNR from +6 dB to +10 dB.

If the training SNR is less than −5 dB the training does not converge, it is too much noise. Above +15 dB for training SNR suffers a relaxing process, there is very little noise. A criterion for fixing the training SNR which produces the greatest output SNR for an input SNR, or for an input SNR range could be set. Hereafter, we will try to find the optimal training SNR with the criterion of having maximum output SNR with low input SNR; furthermore, the output SNR will be greater than the input SNR for all the range. Figure 14 shows the output SNR for training SNR from +6 dB to +10 dB in 1 dB steps. With the mentioned criterion, the optimal training SNR is +7 dB.

### 3.6. Summary of the Design

The objective is to design an equalizer using a TDNN for:

- 1 kbit/s data binary rate,
- the signal received is unipolar NRZ with AWGN,
- 10 samples per bit are taken, so the sample frequency is 10 kHz.

The TDNN has been designed with the following parameters:

- +7 dB for the training SNR,
- a observation interval of 10 samples,
- one hidden layer with one neuron,
- Linear Transfer Function in the hidden layer,
- one neuron in the output layer,
- Satlin Transfer Function for the output neuron,
- initially, 1,000 bits were used (80% for training, 10% validation and 10% for testing),
- conjugate gradient backpropagation with Polak-Ribiere updates training algorithm was used,
- Mean Squared Error function for training.

Finally the TDNN was checking:

- with testing SNR from −5 dB to +20 dB in 0.5 dB steps,
- 1,000 bits were simulated for each SNR value.

The solution obtained was subjected to a second round of parameter variations to see if the solution obtained is local. The sweep was done in the same and reverse order, that is, if changing a parameter can improve the system. By varying the parameters the same solution was obtained.

### 3.7. Stability Band

The TDNN architecture with associated parameters has been reached, but each time it is trained different coefficients are obtained (weights and bias), these coefficients produce similar but not identical curves. That is, variations may occur between TDNNs obtained with the same training SNR. This is because each training session has different initialization of the algorithm. In addition, every training session uses different data and noise signals; although, the values of its parameters: power, statistics features, *etc.*, are held. The stability band can be defined as the zone where it can be ensured that most TDNN curves fit.

**Figure 15.**
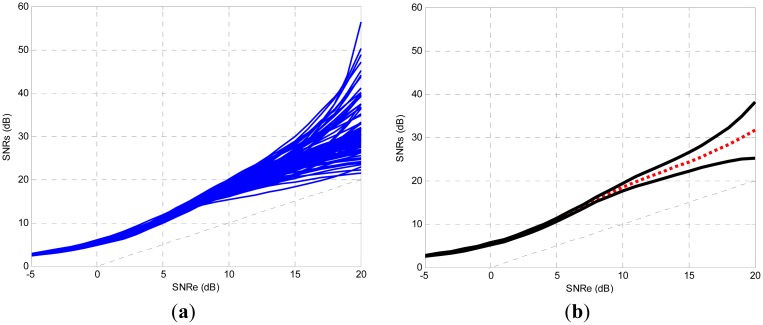
(**a**) Testing curves of successful training. (**b**) Mean value and stability band boundaries.

For this purpose, 100 training sessions were realized. The curves whose SNR output was less than input SNR were neglected. There were 94 successful training sessions; in Figure 15a the 94 testing curves can be observed. Then, for each value of the input SNR the mean value of output SNR was calculated, and the mean value curve is shown in Figure 15b with a dotted line. Furthermore, the typical deviation of the output SNR was calculated for each value of the input SNR. A lower boundary was defined with these values, by subtracting the typical deviation from the mean value. Similarly, an upper bound was defined, adding the typical deviation to the mean value. In Figure 15b the stability band is shown, with its boundaries shown in black line. Of the curves 76% (71 of 94) fell within the stability band.

## 4. The Fixed Point Modelling with System Generator

The calculation that performs a neural network can be fully or partly sequentialized, less hardware resources implies more time delay. Furthermore, the system can be completely parallelized, and the maximum amount of resources entail minimum delay. The level of parallelization chosen depends on the maximum delay allowed and the hardware resources available for the design. For high sample rates the fully parallelized architecture must be used for reducing the response time.

**Figure 16.**
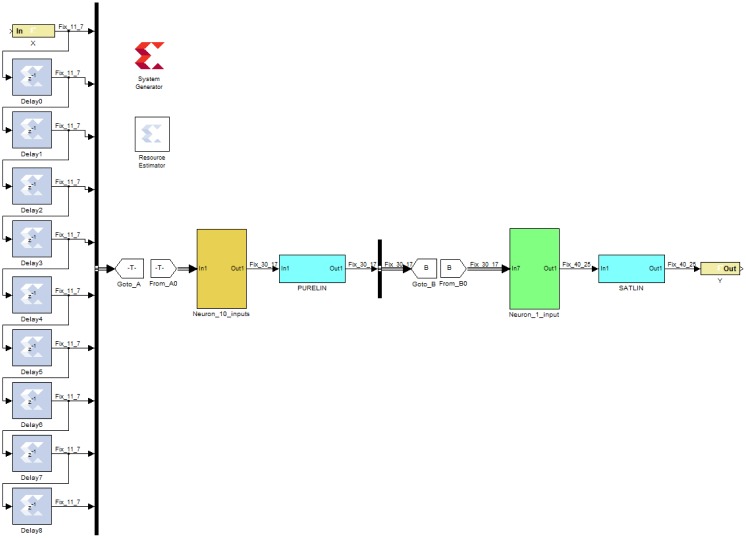
System Generator block diagram of the TDNN.

The TDNN was designed in fixed point format twos complement for an FPGA using the Xilinx System Generator. The Simulink block diagram of the TDNN is shown in Figure 16. From left to right and top to bottom, the figure shows: the FPGA input port, the nine delay elements, the System Generator block, the Resource Estimator block, the first neuron and its transfer function, the second neuron and its transfer function, and the FPGA output port.

Henceforth, the System Generator blocks used are described. Gateway In block is the input bus to the FPGA, the signals would come from an Analog to Digital Converter. If the noise power was zero, the signal would be 0 or +1 and it would be enough an unsigned bit for the representation; in that case, an equalizer would not be necessary. The noisy signal is bipolar and needs a sign bit. The range of the input signal must be covered for the worst case of −5 dB of input SNR. In that situation the noise power (σ^2^) is 1.58, and the noise typical deviation is σ = 1.26. The values *n* in a Gaussian probability density function are in 99.7% within three typical deviations from the mean (Equation (1)):

(1) Pr(μ−3σ≤n≤μ+3σ)=0.997

Then the samples are found with a probability greater than 99.7% between the limits given by Equation (2), and the input signal must be representable in the interval [−3.78, +4.78]:

(2) $$\begin{array}{c} \text{Lower limit}=0-3\sigma =-3.78;\\ \text{Upper limit}=1+3\sigma =+4.78 \end{array}$$

To set the number of decimal bits (*ndb*), the quantization interval (*Δ*) was set as a fraction (*p*) of the peak to peak value of the input signal (Equation (3)), where A is equal to +1 and the maximum quantization error is *nq* = *Δ*/*2*. The quantization interval (*Δ*) was set as a fraction (*p*) of the peak to peak value of the input signal to set the number of decimal bits (*ndb*). This is given by Equation (3), where A is equal to +1 and the maximum quantization error is *Δ*/*2*. For example, the *p* value is 0.01 then the number of decimal bits is 7:

(3) $$\Delta =2^{-\text{ndb}}\leq \text{p}\cdot (\text{A}-0)$$

Nine delay cells are used, each of them delay a clock cycle, the frequency of this clock is the sampling frequency. The 10 samples of the input signal are grouped together using a Bus Creator Simulink block which facilitates the connection to the next stage. Afterwards, Goto and From Simulink blocks are used for wiring. This technique is useful in complex systems with many neurons, where there are many electrical connections.

**Figure 17.**
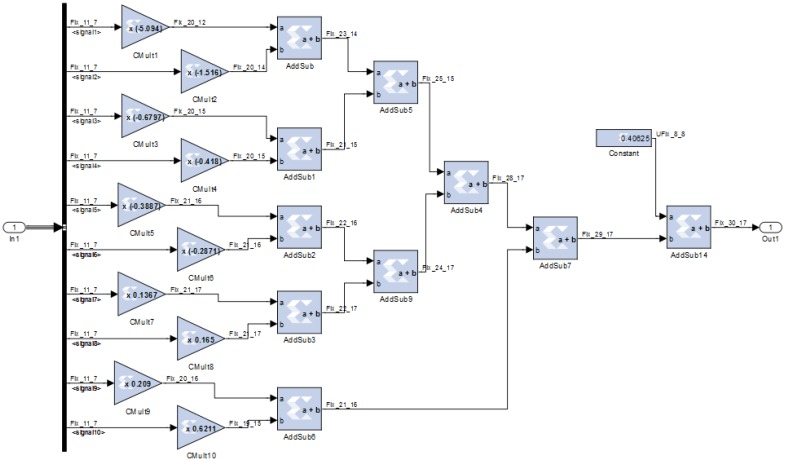
First stage of the hidden layer.

The hidden layer is formed by a single neuron. In the first stage the delay cells outputs are multiplied by weights and the bias value is added. The second stage is the transfer function. Figure 17 shows the first stage. In constant multipliers blocks (Cmult) are specified: the value of the constant, the number of bits for its representation, the binary point position, and the type of output precision. Latency can also be fixed, that is, the number of clock cycles for the multiplication. For increasing the speed this value is set to zero, so the constant multipliers are fully combinational.

Coefficient values are given from a Matlab array in floating point format, these values come from the training process. The number of bits and binary point position are set for covering the range and for representing the value with a maximum error. Initially, the output precision for the constant multipliers was set as full. In the same way, the adders are configured with zero latency and full output resolution. Finally, the bias is added, this value is stored in a Constant block and configured similarly to the constant multiplier block.

The transfer function for this neuron is the identity function (Linear Transfer Function in Matlab). It could be avoided joining the blocks with a connection. A block with an internal connection is used to remember that in other cases it must be inserted a block that performs the transfer function. Obviously, this does not penalize in area, power consumption or delay time.

**Figure 18.**
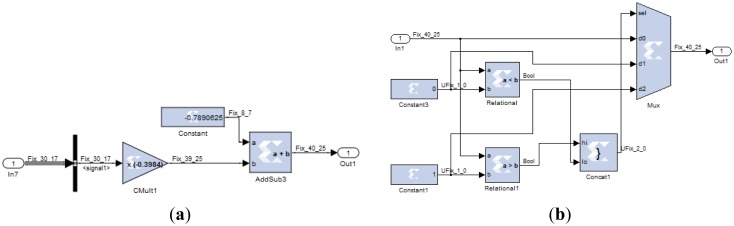
(**a**) First stage of the output layer; (**b**) Transfer function in the output layer.

The output of the hidden layer is connected to the output layer, which has only one neuron. The first stage in Figure 18a is similar to the neuron in the hidden layer, the blocks are configured in the same way. The transfer function is the Satlin Matlab type. It was designed mainly with two comparators and a multiplexer as shown in Figure 18b.

The output precision is set by the multiplexer. Initially, it was set to full resolution. The input from the previous stage is signed; for this reason, the multiplexer has a signed output. Actually, Satlin function output is unsigned, between 0 and +1, it could be eliminated the bit sign, this adjustment was done later.

**Figure 19.**
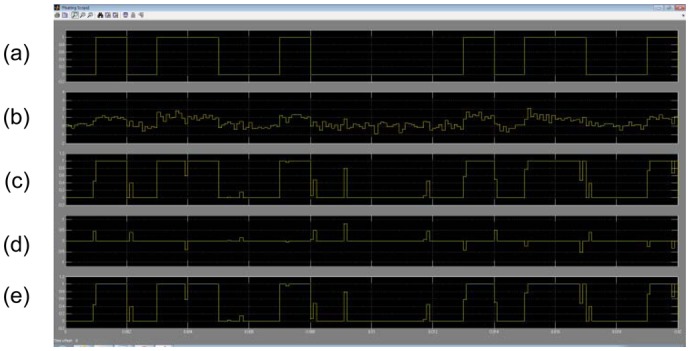
Simulink simulation of the TDNN: (**a**) original data, (**b**) data with noise, (**c**) the output of the FPGA, (**d**) the error signal in the FPGA and (**e**) the output of the TDNN in floating point format.

Under these conditions, the coefficients (weights and bias) are represented with a maximum error of 1%. The peak to peak value (*p*) was set at 1% for the error in the input signal representation. The output was left in full resolution. The TDNN trained with +7 dB for the SNR was simulated on the FPGA with +7 dB testing input SNR. The Simulink simulation is shown in Figure 19.

**Figure 20.**
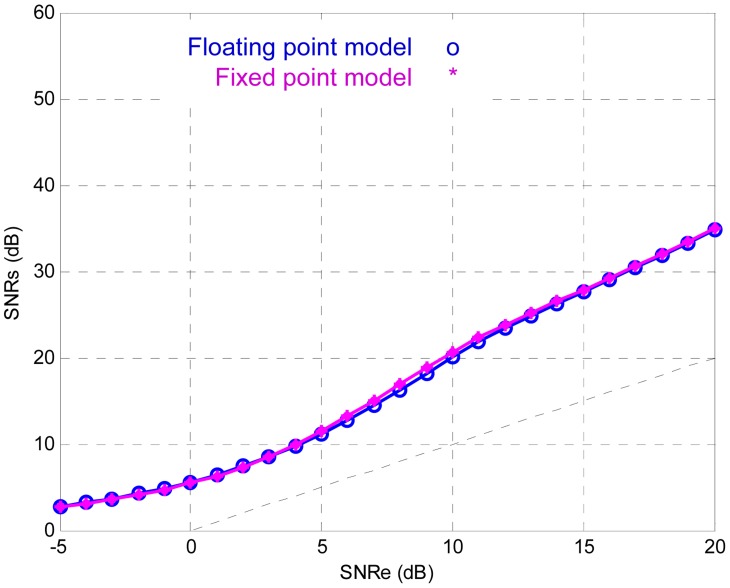
Output SNR *versus* input SNR for the TDNN in floating point format and fixed point format (FPGA).

Finally, the testing SNR was varied from −5 dB to +25 dB in 1 dB increments, for each testing SNR 1,000 random bits were simulated. Figure 20 shows the output SNR *versus* input SNR for the TDNN in floating point format and the TDNN on FPGA in fixed point format. At this point, the functionality of the equalizer on the FPGA has been fully verified.

The number of bits can be reduced for the representation of weights, bias and input samples; this implies saving area, power and less delay time. Decreasing the number of bits produces growth in the representation error and the performance of the system gets worse. In other words, the reduction of number of bits causes degradation of output SNR *versus* input SNR curve. The Table 1 shows the results for 0.1%, 1% and 2% errors. It also shows the optimized model for 1%, which will be explained later. The TDNN in floating point format was trained with +7 dB of SNR, this model for +7 dB of testing SNR causes +14.52 dB for output SNR.

The maximum error must be set through some criterion. For example, that the curve of fixed point model must deviate less than 1 dB respect to the curve of floating point model. With this criterion, the maximum error was set to 1%. In this case, the input signal format has 11 bits: a bit for the sign, three integer bits and seven decimal bits. With full resolution in all operators the output of the TDNN in the FPGA has 40 bits, including 25 decimal bits and a sign bit. Then some adjustments are possible in the output format—this corresponds to the optimized model. First, the sign bit can be removed in the output. Moreover, the number of decimal bits can be reduced to seven without degrading the SNR curve, this can be checked experimentally. Finally, one bit for the integer part is used, so +1 can be represented without error. Given this reduction in the number of bits in the output signal, the number of bits of different stages is reduced in the system from the output to the input.

## 5. Obtaining the Physical Performance with Integrated System Environment

At this point the model and the architecture of the system have been fixed; besides, the full functionality has been checked. Then the design is compiled with System Generator. For the compilation process the FPGA device must be chosen, in this case the Xilinx Spartan-3E family, device xc3s500e, package fg320, and −5 for speed grade was used. Besides, for System Generator compilation a standard Hardware Description Language (HDL) must be chosen, these languages are Verilog and Very High Speed Integrated Circuit Hardware Description Language (VHDL) [21,22]. After the compilation a project is generated for the Xilinx Integrated System Environment (ISE), which includes the HDL files for the structural description of the system.

**Figure 21.**
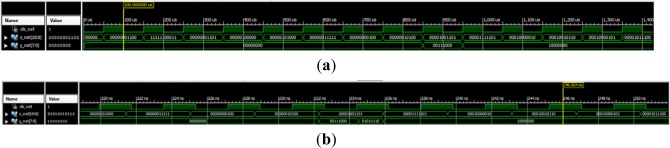
Xilinx ISE timing simulations: (**a**) 1 kbit/s and 10 ksamples/s, (**b**) 27.6167 Mbit/s and 276.167 Msamples/s.

The ISE software is the Xilinx standard tool for FPGA design. The syntax of the HDL files can be checked, and synthesis and behavioral simulation of the TDNN can be executed. After that, the design implementation permits the timing simulation of the system. The simulation for 1 kbit/s is illustrated in Figure 21a. Finally, the programming file is generated for the chosen device.

Xilinx ISE software manages FPGA circuits with a high level of detail. For this reason, the physical performances can be determined more accurately. Table 2 shows these results in area, power and maximum clock frequency for both hardware description languages. The program can even estimate the operating temperature of the circuit. Slight differences were obtained between the standard languages. According to the maximum clock frequency, with this device it would be possible to reach up to 27.6167 Megabits per second, this simulation is in Figure 21b.

It should be emphasized that ISE simulator uses FPGA circuits with high level detail, this makes simulations more accurate, but much slower. Only short duration signals can be simulated, in opposition to System Generator simulations. In this environment the full functionality of the system cannot be tested, but timing details can be analysed.

## 6. Conclusions and Future Lines of Research

A design methodology of an equalizer is presented using a neural network on a FPGA. Three phases can be differentiated in the design, the first two phases are supported by Matlab. In the first stage the Matlab Neural Network Toolbox is used for fixing the floating point architecture, parameters and the performance of the neural network, the information obtained can be called the “golden rule”.

In the second stage the Xilinx System Generator is used, which operates on Matlab Simulink. In this phase the system is designed in fixed point format according to the golden rule. In System Generator the circuits are handled with low level of detail, for this reason the simulations are very fast and the functionality of the system can checked completely. During this stage a poor estimation of the area is calculated, and nor power consumption nor speed of the circuit are evaluated. Moreover, the effect of the number of bits in different parts of the design can be tested. The fixed point format has implications on the functionality of the system and the hardware resources occupied.

In the third step, the system description obtained with System Generator is used by the Xilinx Integrated System Environment. This design tool uses a high level of circuit details, and this allows estimation of physical performances: hardware resources, power consumption and maximum clock frequency.

It should be noted that the description of the system obtained by System Generator is not portable to other manufacturers. The reason is that System Generator calls primitives and specific blocks of Xilinx. The design could have been done for Altera, the second FPGA manufacturer in importance. Altera offers DSP Builder, which is a similar tool over Simulink. In the same way these designs are only valid for Altera FPGAs. As a future line of investigation, Matlab HDL Coder could be used, whose files are portable to all manufacturers. The HDL Coder designs can be compared with the designs obtained with the FPGA manufacturers' tools. The results will depend on the compilers. Provide the portability using a hand coded hardware description language is not a good alternative. The design of complex systems directly in a hardware description language is long and tedious, and not flexible for changes.

Obviously, increasing the sampling frequency can improve the system performance, but this may be a design restriction or be limited by the technology. In other words, given the sampling frequency it is possible to improve the system by varying other parameters of the neural network.

The output SNR curve obtained is not rigid, and among other parameters it depends on the architecture and training SNR. The neural network can be trained for other scenarios, for instance if the signal suffers from distortion or other noise model. Another advantage is that the architecture and parameters can be changed to fit the new channel. Other neural network architectures are available, even some of them with feedback signals.

The same error was assumed in the representation of the input and coefficients in the two layers. In general the effect of different errors should be analysed for input and each layer coefficients. The conclusions should focus on the functionality and physical performance of the system. This study should be automated with a Matlab program for executing the models designed with System Generator. In this case, given the shape of the transfer functions its effect is not considered. That is, these functions do not produce approximation errors between input and output. In general, with other nonlinear functions it is necessary to consider the approximation error introduced by the implementation.

During system design it is convenient to maintain full the resolution of the operators. Reducing the number of bits sometimes is possible in the final output. In this case it is possible to decrease the binary representation toward the circuit inputs. This process could also be automated with a Matlab program. The low rates used in the initial simulations do not affect the method or the conclusions, being generalizable to higher frequencies, as high as allowed by the available technology.

## Figures and Tables

**Table 1.** Effect of the errors on the system.

**Error**	**SNR_output_** **(SNR_input_= +7dB)**	**SNR_output_*versus*** **SNR_input_**	**Difference between** **Fixed Point and** **Floating Point** **Models**	**AREA**	**Input** **Format**	**Output** **Format**
		Floating point: o				
		Fixed point:*				
0.1%	14,47 dB	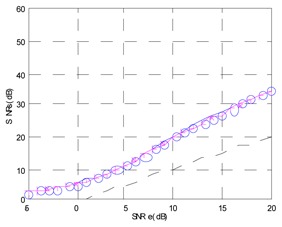	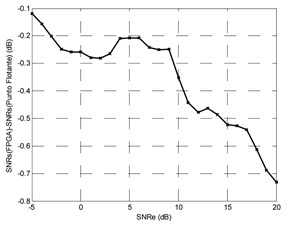 Worst case: −0.7 dB	Slices: 1026 FFs: 126 BRAMs: 0 LUT: 1869 IOBs: 64 Mults/DSP48s: 0 TBUFs: 0	Signed Number of bis: 14 Binary point: 10	Signed Number of bis: 50 Binary point: 35
1%	14,47 dB	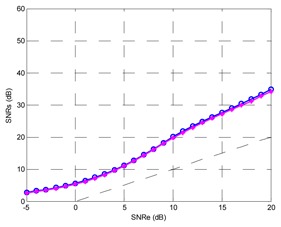	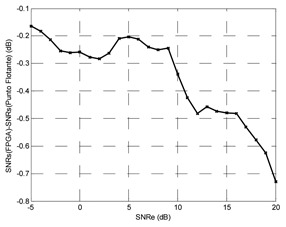 Worst case: −0.7 dB	Slices: 693 FFs: 99 BRAMs: 0 LUT: 1238 IOBs: 51 Mults/DSP48s: 0 TBUFs: 0	Signed Number of bis: 11 Binary point: 7	Signed Number of bis: 40 Binary point: 25
1% (optimized)	14,46 dB	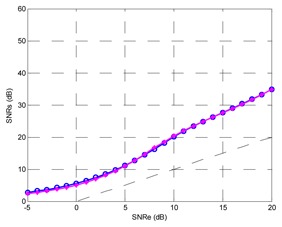	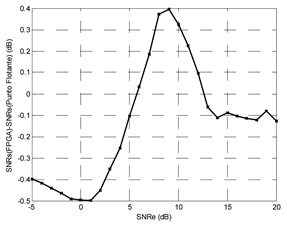 Worst case: −0.5 dB	Slices: 678 FFs: 99 BRAMs: 0 LUT: 1203 IOBs: 19 Mults/DSP48s: 0 TBUFs: 0	Signed Number of bis: 11 Binary point: 7	Unsigned Number of bis: 8 Binary point: 7
2%	14,46 dB	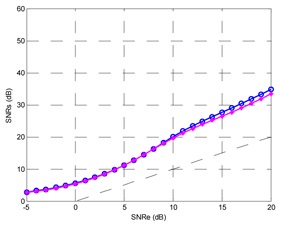	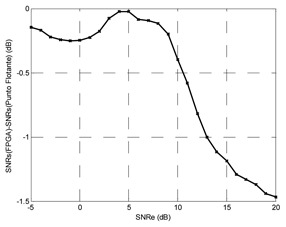 Worst case: −1.5 dB	Slices: 623 FFs: 90 BRAMs: 0 LUT: 1106 IOBs: 47 Mults/DSP48s: 0 TBUFs: 0	Signed Number of bis: 10 Binary point: 6	Signed Number of bis: 37 Binary point: 22

**Table 2.** Results in area, power and maximum clock frequency for both HDL.

**HDL**	**Area**	**Power (W)**	**Maximum Clock Frequency**
VHDL	Number of Slices: 602 out of 4656: 12%	Quiescent: 0.085 Dynamic: 0.292 Total: 0.377	276.167 MHz
	Number of Slice Flip Flops: 100 out of 9312: 1%		
	Number of 4 input LUTs: 961 out of 9312: 10%		
	Number of IOs: 21		
	Number of bonded IOBs: 20 out of 232: 8%		
	Number of GCLKs: 1 out of 24: 4%		
Verilog	Number of Slices: 602 out of 4656: 12%	Quiescent: 0.085 Dynamic: 0.292 Total: 0.377	273.523 MHz
	Number of Slice Flip Flops: 100 out of 9312: 1%		
	Number of 4 input LUTs: 963 out of 9312: 10%		
	Number of IOs: 21		
	Number of bonded IOBs: 20 out of 232: 8%		
	Number of GCLKs: 1 out of 24: 4%		

## References

[1] Duda R.O., Hart P.E., Stork D.G. (2001). Pattern Classification.

[2] Sklar B. (1998). Digital Communications. Fundamentals and Applications.

[3] Matlab. http://www.mathworks.com/products/.

[4] Hauck S., DeHon A. (2008). Reconfigurable Computing.

[5] System Generator. http://www.xilinx.com/tools/sysgen.htm.

[6] Simulink. http://www.mathworks.com/help/simulink/.

[7] Integrated System Environment. http://www.xilinx.com/products/design-tools/ise-design-suite/index.htm.

[8] Watterson J.W. (1990). An optimum multilayer perceptron neural receiver for signal detection. IEEE Trans. Neural Netw..

[9] Feng J., Tse C.K., Lau F.C.M. (2003). A neural-network-based channel-equalization strategy for chaos-based communication systems. IEEE Trans. Circuits Syst. I Fundam. Theory Appl..

[10] Graham W. (1992). Developments in Non-Linear Equalization. Ph.D. Thesis.

[11] Patra J.C., Pal R.N., Baliarsingh R., Panda G. (1999). Nonlinear channel equalization for QAM signal constellation using artificial neural networks. IEEE Trans. Syst. Man Cybrn..

[12] Aravindan M., Kingsley S.R. Recognition of Modulation Using Multilayer Perceptron in Digital Communication.

[13] Al-Hinai A., Ibnkahla M. Neural Network Nonlinear MIMO Channel Identification and Receiver Design.

[14] Bruyne P., Kjelsen O., Sacroug O. Spread Spectrum Digital Signal Synchronization Using Neural Networks.

[15] Chow T., Feng J.C., Ng K.T. (2000). An adaptive demodulator for the chaotic modulation communication system with RBF neural network. IEEE Trans. Syst. Man Cybrn..

[16] Dickenson R.J., Ghassemlooy Z. (2004). BER performance of 166 Mbit/s OOK diffuse indoor IR link employing wavelets and neural networks. Electron. Lett..

[17] Yen C.T., Weng W., Lin Y.T. (2004). FPGA Realization of a neural-network-based nonlinear channel equalizer. IEEE Trans. Ind. Electron..

[18] Ortiz-Fuentes J.D., Forcada M.L. A Comparison between Recurrent Neural Network Architectures for Digital Equalization.

[19] Yogi S., Subhashini K.R., Satapathy J.K. A PSO Based Functional Link Artificial Neural Network Training Algorithm for Equalization of Digital Communication Channels.

[20] Neural Network Toolbox. http://www.mathworks.com/products/neural-network.

[21] Palnitkar S. (2003). Verilog HDL.

[22] Pedroni V.A. (2004). Circuit Design with VHDL.

